# ERICA: prevalence of common mental disorders in Brazilian adolescents

**DOI:** 10.1590/S01518-8787.2016050006690

**Published:** 2016-02-02

**Authors:** Claudia S Lopes, Gabriela de Azevedo Abreu, Debora França dos Santos, Paulo Rossi Menezes, Kenia Mara Baiocchi de Carvalho, Cristiane de Freitas Cunha, Mauricio Teixeira Leite de Vasconcellos, Katia Vergetti Bloch, Moyses Szklo

**Affiliations:** I Departamento de Epidemiologia. Instituto de Medicina Social. Universidade do Estado do Rio de Janeiro. Rio de Janeiro, RJ, Brasil; II Programa de Pós-Graduação em Saúde Coletiva. Instituto de Medicina Social. Universidade do Estado do Rio de Janeiro. Rio de Janeiro, RJ, Brasil; III Instituto de Medicina Social. Universidade do Estado do Rio de Janeiro. Rio de Janeiro, RJ, Brasil; IV Departamento de Medicina Preventiva. Faculdade de Medicina. Universidade de São Paulo. São Paulo, SP, Brasil; V Departamento de Nutrição. Faculdade de Ciências da Saúde. Universidade de Brasília. Brasília, DF, Brasil; VI Departamento de Pediatria. Faculdade de Medicina. Universidade Federal de Minas Gerais. Belo Horizonte, MG, Brasil; VII Escola Nacional de Ciências Estatísticas. Fundação Instituto Brasileiro de Geografia e Estatística. Rio de Janeiro, RJ, Brasil; VIII Instituto de Estudos em Saúde Coletiva. Universidade Federal do Rio de Janeiro. Rio de Janeiro, RJ, Brasil

**Keywords:** Adolescent, Mental Disorders, epidemiology, Prevalence, Mental Health, Cross-Sectional Studies

## Abstract

**OBJECTIVE:**

To describe the prevalence of common mental disorders in Brazilian adolescent students, according to geographical macro-regions, school type, sex, and age.

**METHODS:**

We evaluated 74,589 adolescents who participated in the Cardiovascular Risk Study in Adolescents (ERICA), a cross-sectional, national, school-based study conducted in 2013-2014 in cities with more than 100,000 inhabitants. A self-administered questionnaire and an electronic data collector were employed. The presence of common mental disorders was assessed using the General Health Questionnaire (GHQ-12). We estimated prevalence and 95% confidence intervals of common mental disorders by sex, age, and school type, in Brazil and in the macro-regions, considering the sample design.

**RESULTS:**

The prevalence of common mental disorders was of 30.0% (95%CI 29.2-30.8), being higher among girls (38.4%; 95%CI 37.1-39.7) when compared to boys (21.6%; 95%CI 20.5-22.8), and among adolescents who were from 15 to 17 years old (33.6%; 95%CI 32.2-35.0) compared to those aged between 12 and 14 years (26.7%; 95%CI 25.8-27.6). The prevalence of common mental disorders increased with age for both sexes, always higher in girls (ranging from 28.1% at 12 years to 44.1% at 17 years) than in boys (ranging from 18.5% at 12 years to 27.7% at 17 years). We did not observe any significant difference by macro-region or school type. Stratified analyses showed higher prevalence of common mental disorders among girls aged from 15 to 17 years of private schools in the North region (53.1; 95%CI 46.8-59.4).

**CONCLUSIONS:**

The high prevalence of common mental disorders among adolescents and the fact that the symptoms are often vague mean these disorders are not so easily identified by school administrators or even by health services. The results of this study can help the proposition of more specific prevention and control measures, focused on highest risk subgroups.

## INTRODUCTION

In recent decades, the patterns of physical and mental illness in children and adolescents have changed considerably. The prevalence of emotional and conduct problems is around 10.0%-20.0%, representing a significant burden of disease, which causes loss in school life and in family and social relationships for these children and adolescents^1^. In addition, mental health problems are highly persistent, causing a significant portion of these individuals to have some impairment in adult life^21^.

A study on the global burden of disease in adolescents and young people aged between 10 and 24 years showed that, worldwide, the three leading causes of years of life lost due to disability in this age group are, respectively, neuropsychiatric disorders (45.0%), unintentional injuries (12.0%) and infectious and parasitic diseases (10.0%)^9^.

In Brazil, a population-based study (Sao Paulo Megacity Mental Health Study)^29^, showed that the average age for the onset of psychiatric disorders is earlier for anxiety disorders (13 years old) and impulse control disorders (14 years old), when compared with substance abuse disorders (24 years old) and mood disorders (36 years old). Another population-based study, conducted in four cities in four regions of Brazil (Southeast, Midwest, Northeast and North)^22^, assessed the prevalence of psychiatric disorders in 1,676 children and adolescents aged from six to 16 years, who attended elementary school, from the second to the sixth year. The overall prevalence for the presence of at least one psychiatric disorder was 13.1%, similar to values found in other population-based studies around the world^3^ ^,^ ^19^.

About 90.0% of mental disorders are non-psychotic disorders^30^. Such disorders due to their high prevalence in the general population (20.0%-30.0%), are usually called common mental disorders (CMD), mainly characterized by the presence of symptoms of depression and anxiety, and various nonspecific and somatic complaints^8^. CMD affect individuals in different age groups and, when present in children and adolescents, may be early and less specific manifestations of more serious mental disorders, also impairing the social relationships and school performance of this population^21^. Early identification of CMD and its main risk factors can contribute to specific interventions and a better prognosis.

In Brazil, some studies evaluated the prevalence and risk factors for CMD in the adult population^13^ ^-^ ^15^ ^,^ ^25^. However, the literature on CMD in adolescents is scarce. We have only identified one cross-sectional, population-based study, conducted among adolescents aged from 15 to 18 years from Pelotas, RS, Southern Brazil, whose CMD prevalence was of 28.8% and the main associated factors were low maternal education, smoking, sedentary behavior, and body image dissatisfaction^23^.

This study aimed to describe the prevalence of common mental disorders in the population of Brazilian adolescent students, according to region, school type, and sociodemographic characteristics.

## METHODS

This study is part of the *Estudo de Riscos Cardiovasculares em Adolescentes* (ERICA – Study of Cardiovascular Risk in Adolescents), a cross-sectional, national, school-based study, conducted in 2013-2014, with the objective of estimating the prevalence of metabolic syndrome, diabetes mellitus, obesity, cardiovascular risk factors, and insulin and inflammatory resistance markers in adolescents aged from 12 to 17 years, who attend schools in cities municipalities with over 100,000 inhabitants.

We assessed 74,589 adolescents in 1,247 schools of 124 Brazilian cities. The research population was stratified into 32 strata comprised of 27 state capitals and five sets of cities with more than 100,000 inhabitants in each geographical macro-region of the Country. For each geographic stratum, we selected schools with probability proportional to size and inversely proportional to the distance to the capital. The sample is representative for the group of cities with more than 100,000 inhabitants at the national, regional and state capitals level. More details on the sampling design can be found in a previous publication^28^. We excluded from the analysis, for not being considered eligible, adolescents outside the age group of 12-17 years, pregnant adolescents and those with physical or mental impairment, temporary or permanent. We have previously described the study protocol^2^.

Data were collected using a self-administered questionnaire, which was entered into the electronic data collector PDA (personal digital assistant). The questionnaire consisted of about 100 questions divided into 11 blocks: sociodemographic aspects, occupational activities, physical activity, eating habits, smoking, alcohol use, reproductive health, oral health, sleep duration, physical morbidity (self-reported) and mental health.

In this study, we analyzed the following characteristics: sex, age, school type (public or private), geographic macro-region (North, Northeast, Midwest, Southeast and South) and presence of CMD. The age variable was used categorically, considering two groups: from 12 to 14 years and from 15 to 17 years.

For the CMD evaluation, we used the General Health Questionnaire, 12-item version (GHQ-12)^6^. The scores of the individual items were coded as “missing” or “present” (0 or 1, respectively) and then summed up; adolescents with a score of three or more were classified as cases of CMD^17^. The Brazilian version, used in this study, was submitted to a validation study, using a structured psychiatric interview as the gold standard and the same criterion of three or more to establish a case of CMD. The results showed a sensitivity of 85.0%, a specificity of 79.0%, and area under the ROC (Receiver Operating Characteristics) curve of 0.87^7^.

We calculated the prevalences and 95% confidence intervals (95%CI) of CMD according to sex, age, and school type, considering the national, regional and capital levels.

The analysis was performed using the Stata 14.0 statistical package. We adjusted the distributions of the features according to the sample design, using statistical routines for complex samples. The ERICA sample is a complex one^26^, since it employs stratification and clustering in its selection stages. We calculated the sample weights using the product of the inverse of inclusion probabilities at each stage of the sample, and calibrated it considering the projection of the number of adolescents enrolled in schools located in geographic areas considered on December 31, 2013. We used a post-stratification estimator, which modifies the natural weight of the design by a calibration factor that is the ratio between the total population and the total estimated by the natural weight of the design for the post-stratum or estimated domain considered.

All participating students signed an assent form and brought the informed consent form signed by their legal guardians (when required by the local Research Ethics Committee). The study was approved by the Research Ethics Committee of the institution of the Study Coordination Center (IESC/UFRJ – Process 45/2008) and by each Brazilian state involved.

## RESULTS

Of the 102,327 eligible adolescents registered in the selected schools, 74,589 (72.9%) participated in the study.

Nearly a third of adolescents from cities with more than 100,000 inhabitants in Brazil presented CMD. The prevalence was higher in the female sex and in older adolescents. The prevalence did not differ by macro-region and school type (Table 1).

**Table 1:** Sample size, population and prevalence of common mental disorders in cities with more than 100,000 inhabitants, according to sex, age, school type and macro-region. ERICA, Brazil, 2013-2014.

Variable	Sample	Estimated population	Prevalence (%)	95%CI
Sex				
Female	41,225	5,052,137	38.4	37.1-39.7
Male	33,364	5,095,563	21.6	20.5-22.8
Age group				
12-14	34,141	5,348,201	26.7	25.8-27.6
15-17	40,448	4,799,499	33.6	32.2-35.0
School type				
Public	58,707	8,382,253	30.0	29.1-31.0
Private	15,882	1,765,447	29.8	28.7-30.8
Macro-region				
North	15,073	855,362	31.3	30.0-32.5
Northeast	23,167	2,165,033	30.2	28.7-31.8
Midwest	9,727	778,010	31.7	30.4-33.0
Southeast	17,080	5,153,506	29.3	28.0-30.7
South	9,542	1,195,789	30.4	28.6-32.2
Brazil	74,589	10,147,700	30.0	29.2-30.8

**Figure 1:**
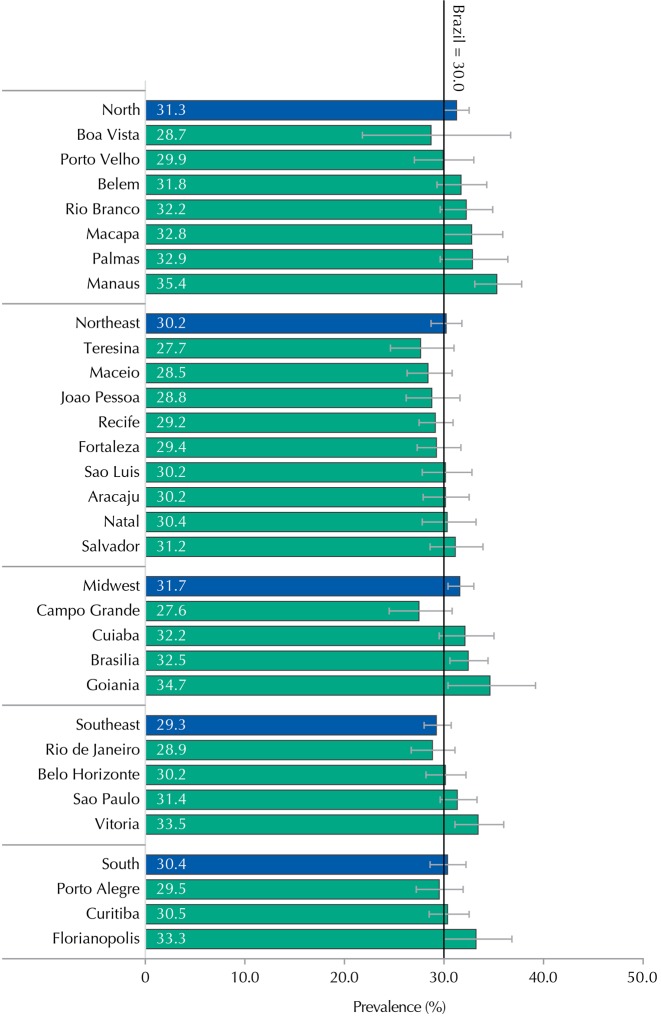
Prevalence and 95%CI of common mental health disorders in the cities with more than 100,000 inhabitants per macro-region and capitals. ERICA, Brazil, 2013-2014.

Regarding the cities, we also found no significant differences, with the highest prevalence of CMD being observed in Manaus, AM, Northern Brazil, and the lowest in Campo Grande, MS, Midwestern Brazil (Figure 1).

**Figure 2:**
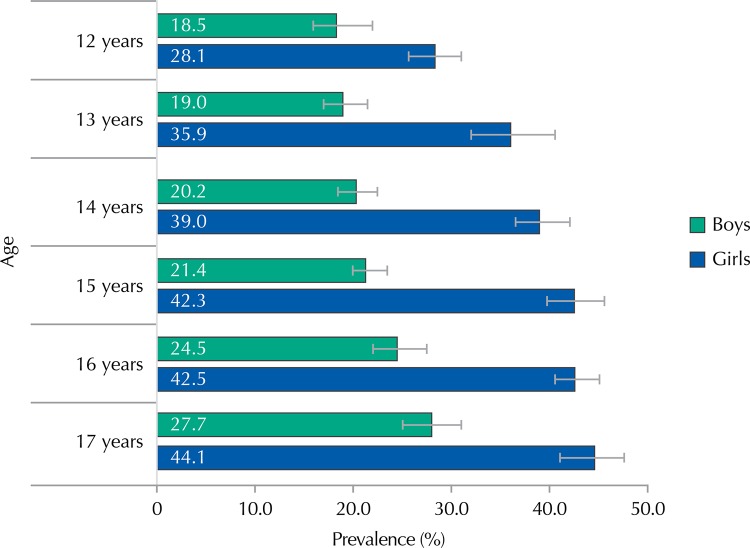
Prevalence (%) and 95%CI of common mental disorders in the cities with more than 100,000 inhabitants by sex and age. ERICA, Brazil, 2013-2014.

Prevalence of CMD among girls was always higher than among boys, for all age groups (Figure 2). We also observed a trend of increasing CMD prevalence as age increased.

In the analysis stratified by sex, age, school type and macro-region, we observed that the highest prevalence of CMD was among girls, aged from 15 to 17 years, and from private schools in the North region. The lowest prevalence was among boys, aged from 12 to14 years, from private schools in the Southeast region (Table 2).

**Table 2:** Prevalence (%) and 95%CI of common mental disorders in adolescents by macro-region, according to the school type, sex and age group. ERICA, Brazil, 2013-2014.

Variable	Brazil		Macro-region									

			North		Northeast		Midwest		South		Southeast	
					
	%	95%CI	%	95%CI	%	95%CI	%	95%CI	%	95%CI	%	95%CI
Public school												
Girls												
12-14 years	33.8	31.9-35.9	35.0	32.2-37.8	29.8	26.5-33.3	35.9	33.1-38.9	31.8	26.8-37.3	35.3	32.0-38.7
15-17 years	42.3	40.0-44.6	44.9	43.1-46.8	42.5	40.0-45.1	44.7	42.1-47.4	40.7	36.4-45.1	41.7	37.6-46.0
Boys												
12-14 years	20.0	17.9-22.2	18.8	16.9-20.8	23.6	16.1-33.1	19.3	15.5-23.7	23.2	17.5-29.9	18.3	16.2-20.6
15-17 years	24.0	22.4-25.6	25.4	23.3-27.6	24.5	21.8-27.4	25.8	23.5-28.3	26.7	22.7-31.2	22.6	20.0-25.3

Total	30.0	29.1-31.0	30.9	29.6-32.3	30.4	28.4-32.3	31.3	29.9-32.8	30.5	28.5-32.5	29.4	27.8-31.0

Private school												
Girls												
12-14 years	36.4	33.3-39.5	39.1	33.0-45.5	35.3	32.4-38.3	38.2	34.2-42.4	32.8	26.5-39.8	37.2	31.7-43.1
15-17 years	46.5	42.6-50.5	53.1	46.8-59.4	47.4	43.8-50.9	44.6	39.4-49.9	42.1	38.0-46.3	46.7	39.7-53.9
Boys												
12-14 years	16.3	14.6-18.2	20.4	15.6-26.2	18.1	15.4-21.2	20.0	14.3-27.1	21.5	15.5-29.0	13.0	11.0-15.4
15-17 years	26.0	24.0-28.1	32.3	28.7-36.1	27.2	24.0-30.8	34.1	28.7-40.0	26.7	22.1-31.9	23.8	21.3-26.5

Total	29.8	28.7-30.8	35.0	31.6-38.6	29.7	27.9-31.6	33.3	30.8-36.0	29.6	26.5-32.8	28.8	27.2-30.4

## DISCUSSION

This is the first epidemiological study conducted in Brazil, with representation for the cities with 100,000 inhabitants or more on a national level, which evaluated mental morbidity data in adolescents.

The overall prevalence of these disorders was high, being higher among older girls (15-17 years) in private schools in the North region, followed by those from public schools in the same region. The general prevalences by macro-regions and by school type, however, showed no significant differences.

Although we do not have mental health data of Brazilian adolescents with national representation, the overall prevalence of CMD found in ERICA (30.0%) was similar to that observed in a population-based study conducted among adolescents aged from 15 to 18 years living in Pelotas, a medium-sized city in Southern Brazil (28.8%)^23^. The higher prevalence of CMD in girls (38.4%) when compared to boys (21.6%) found in this study is also consistent with the findings of the Pelotas study, in which the prevalence of CMD among girls was 37.2% and among boys was 19.9%. A Brazilian study, conducted in four cities of four regions of Brazil (North, Northeast, Midwest and Southeast) found an overall prevalence of 13.1% (in the last 12 months) for at least one psychiatric disorder^22^. This prevalence is much lower than the one found in our study and probably reflects some differences between the two studies, namely: 1) the ERICA study used a screening instrument for mental disorders (GHQ-12), while the study by Paula et al.^22^ applied a tool for psychiatric disorders diagnosis (Schedule for Affective Disorders and Schizophrenia for School-Age Children – K-SADS-PL); 2) in ERICA, the age group of participants was 12-17 years, while in the study by Paula et al., participants were aged between six and 16 years; and 3) ERICA is a study with national representativeness, conducted among 75,000 students, while in the study by Paula et al., the sample remained restricted to only four cities. In addition, the study by Paula et al. observed a higher prevalence (18.5%) of psychiatric disorders (any disorder) in the city located in the Midwest region. In this study we found differences not really significant in CMD prevalence by macro-regions, with it being slightly higher only in the Midwest and North regions.

Comparisons with international studies are even more difficult, due to differences in methods, in types of disorders and in age groups employed in the studies. However, a review of the results from population-based surveys conducted in different parts of the world shows that, despite the substantial variation in the results, approximately one quarter of adolescents experienced some mental disorder in the previous year and a third during their lifetimes^18^. Studies have also been consistent in pointing higher prevalence of anxiety and mood disorders among girls, while boys have higher rates of behavior and conduct disorders^19^ ^,^ ^20^ ^,^ ^24^,varying according to income and countries’ development . Conduct disorders, communications disorders, and pervasive developmental disorders are more common in the early stage of childhood, while in adolescence conduct disorders and depression prevail^20^ ^,^ ^24^.

Studies show a higher prevalence of disorders among adolescents living in disadvantaged countries or regions; however, the findings are inconsistent, likely reflecting differences in the used methods. Giel et al.^5^ (1981) found a prevalence of mental disorders ranging from 12.0% to 29.0% in children and adolescents aged from five to 15 years in four countries with low or average income (Sudan, Philippines, Colombia and India). A study conducted in Oman, in Arabia, with 5,409 adolescents and young adults of both sexes, aged between 14 and 23 years, found a prevalence of 20.0% for at least one psychiatric diagnosis, according to the criteria of the Diagnostic and Statistical Manual of Mental Disorders, Fourth Edition (DSM-IV), and 32.7% for mild mental disorders (any disorder in the last 12 months)^11^. Thabet and Vostanis^27^ (1998) reported a 21.0% prevalence of anxiety symptoms and related disorders among children living in the Gaza Strip, comparable to data found by Kashani and Orvaschel^12^ (1990) in the population of children and adolescents of USA. In India, a recent meta-analysis on the prevalence of mental disorders in children and adolescents showed that among schoolchildren from five to 15 years, the prevalence of psychiatric disorders was 23.3%^16^. These prevalence rates are lower than those found in this study that used an instrument for CMD assessment and non-psychiatric diagnoses, like the studies mentioned above.

This is the first study that evaluated data of physical and mental health of in adolescent students, with representation for cities with more than 100,000 inhabitants, for Brazil, macro-regions, and state capitals. CMD were evaluated by standardized instrument and validated for the population of children and adolescents^4^, allowing comparisons with national and international studies. However, we should consider that the GHQ-12 is a screening instrument sensitive to recent psychological changes, leading to a greater number of false positives, with transient symptoms of psychological disorders, thus overestimating the prevalence of CMD. In addition, more detailed socioeconomic information was not included in the analysis and it should be considered in future ones.

Mental disorders have emerged as major challenges health services must face. Often, before the formal diagnosis of a psychiatric disorder, it is already possible to find evidence of psychic suffering in adolescents during clinical practice. Therefore, early identification of CMD and its main risk factors can help proposals for preventive and more specific control measures over the entire adolescent development process.

This study, part of a larger study whose main objective is to investigate cardiovascular risk factors in adolescents, included, in addition to mental health, the measurement of a number of factors such as sociodemographic characteristics, lifestyle, referred morbidity and anthropometric and biochemical data (on a subsample). Thus, we expect that the results of this study, which show high prevalence of CMD in children and adolescents, will provide important evidence for future studies that shall evaluate common mechanisms underlying mental disorders and cardiovascular diseases; and, on a longitudinal perspective, shal investigate the role of cardiovascular disease in the occurrence and persistence of mental disorders. Such studies will allow a better understanding of this comorbidity, enabling the development of effective treatments and interventions^10^.
