# Supplementary material for: ERICA: prevalence of common mental disorders in Brazilian adolescents
Source: Rev Saude Publica. 2016 Feb 2;50(Suppl 1):14s. doi: 10.1590/S01518-8787.2016050006690 (PMC4767030; doi:10.1590/S01518-8787.2016050006690)
Supplement: Translation

# Translation: ERICA: prevalência de transtornos mentais comuns em adolescentes brasileiros

**Authors:** Claudia S Lopes, Gabriela de Azevedo Abreu, Debora França dos Santos, Paulo Rossi Menezes, Kenia Mara Baiocchi de Carvalho, Cristiane de Freitas Cunha, Mauricio Teixeira Leite de Vasconcellos, Katia Vergetti Bloch, Moyses Szklo

**Affiliations:** I Departamento de Epidemiologia. Instituto de Medicina Social. Universidade do Estado do Rio de Janeiro. Rio de Janeiro, RJ, Brasil; II Programa de Pós-Graduação em Saúde Coletiva. Instituto de Medicina Social. Universidade do Estado do Rio de Janeiro. Rio de Janeiro, RJ, Brasil; III Instituto de Medicina Social. Universidade do Estado do Rio de Janeiro. Rio de Janeiro, RJ, Brasil; IV Departamento de Medicina Preventiva. Faculdade de Medicina. Universidade de São Paulo. São Paulo, SP, Brasil; V Departamento de Nutrição. Faculdade de Ciências da Saúde. Universidade de Brasília. Brasília, DF, Brasil; VI Departamento de Pediatria. Faculdade de Medicina. Universidade Federal de Minas Gerais. Belo Horizonte, MG, Brasil; VII Escola Nacional de Ciências Estatísticas. Fundação Instituto Brasileiro de Geografia e Estatística. Rio de Janeiro, RJ, Brasil; VIII Instituto de Estudos em Saúde Coletiva. Universidade Federal do Rio de Janeiro. Rio de Janeiro, RJ, Brasil

**Keywords:** Adolescente, Transtornos Mentais, epidemiologia, Prevalência, Saúde Mental, Estudos Transversais

## Abstract

**OBJETIVO:**

Descrever a prevalência de transtornos mentais comuns em adolescentes escolares brasileiros, segundo macrorregiões, tipo de escola, sexo e idade.

**MÉTODOS:**

Foram avaliados 74.589 adolescentes participantes do Estudo de Riscos Cardiovasculares em Adolescentes (ERICA), estudo transversal, nacional, de base escolar, realizado em 2013-2014 em municípios com mais de 100 mil habitantes. Utilizou-se questionário autopreenchível e coletor eletrônico de dados. Presença de transtornos mentais comuns foi avaliada por meio do General Health Questionnaire (GHQ-12). Estimaram-se prevalências e intervalos de confiança de 95% de transtornos mentais comuns por sexo, idade e tipo de escola, no Brasil e nas macrorregiões, considerando o desenho da amostra.

**RESULTADOS:**

A prevalência de transtornos mentais comuns foi de 30,0% (IC95% 29,2-30,8), sendo mais elevada entre meninas (38,4%; IC95% 37,1-39,7), quando comparadas aos meninos (21,6%; IC95% 20,5-22,8) e entre os adolescentes de 15 a 17 anos (33,6%; IC95% 32,2-35,0), em relação àqueles entre 12 e 14 anos (26,7%; IC95% 25,8-27,6). As prevalências de transtornos mentais comuns aumentaram conforme a idade, para ambos os sexos, sempre maior nas meninas (variando de 28,1% aos 12 anos, até 44,1% aos 17 anos), do que nos meninos (variando de 18,5% aos 12 anos até 27,7% aos 17 anos). Não houve diferença importante por macrorregião ou tipo de escola. Análises estratificadas mostraram maior prevalência de transtornos mentais comuns entre meninas de 15 a 17 anos de escolas privadas da região Norte (53,1; IC95% 46,8-59,4).

**CONCLUSÕES:**

A elevada prevalência de transtornos mentais comuns entre os adolescentes e o fato de os sintomas serem muitas vezes vagos fazem com que esses transtornos sejam pouco identificados por gestores escolares ou mesmo serviços de saúde. Os resultados deste estudo podem ajudar na proposição de medidas de prevenção e controle mais específicas e voltadas para os subgrupos sob maior risco.

## INTRODUÇÃO

Nas últimas décadas, os padrões de adoecimento físico e mental de crianças e adolescentes têm mudado consideravelmente. A prevalência de problemas emocionais e de conduta é em torno de 10,0%-20,0%, constituindo uma carga de doença expressiva, com prejuízo na vida escolar e nas relações familiares e sociais dessas crianças e adolescentes^1^ ^.^ Além disso, problemas de saúde mental são altamente persistentes, fazendo com que parcela importante desses indivíduos tenha algum prejuízo na vida adulta^21^ ^.^

Estudo sobre a carga global de doenças em adolescentes e jovens entre 10 e 24 anos mostrou que, mundialmente, as três principais causas de anos de vida perdidos por incapacidade nessa faixa etária são, respectivamente, os transtornos neuropsiquiátricos (45,0%), as lesões não intencionais (12,0%) e as doenças infecciosas e parasitárias (10,0%)^9^ ^.^

No Brasil, estudo de base populacional conduzido em São Paulo (*Sao Paulo Megacity Mental Health Study*)^29^ mostrou que a idade média de início de transtornos psiquiátricos é mais precoce para os transtornos de ansiedade (13 anos de idade) e transtornos do controle de impulsos (14 anos de idade), quando comparados aos transtornos de abuso de substâncias (24 anos de idade) e transtornos do humor (36 anos de idade). Outro estudo de base populacional, conduzido em quatro cidades de quatro regiões do Brasil (Sudeste, Centro-Oeste, Nordeste e Norte)^22^ ^,^ avaliou a prevalência de transtornos psiquiátricos em 1.676 crianças e adolescentes escolares de seis a 16 anos que cursavam do segundo ao sexto ano do ensino fundamental. A prevalência total para a presença de pelo menos um transtorno psiquiátrico foi de 13,1%, semelhante àquela encontrada em outros estudos de base populacional no mundo^3^ ^,^ ^19^ ^.^

Cerca de 90,0% dos transtornos mentais compõem-se de transtornos não psicóticos^30^ ^.^ Tais transtornos, em virtude de sua elevada prevalência na população geral (20,0%-30,0%), são usualmente denominados transtornos mentais comuns (TMC), caracterizados principalmente pela presença de sintomas de depressão e ansiedade, além de diversas queixas inespecíficas e somáticas^8^ ^.^ Os TMC afetam indivíduos em diferentes faixas etárias e, quando presentes em crianças e adolescentes, podem ser manifestações iniciais e menos específicas de transtornos mentais mais graves, além de causarem prejuízo nas relações sociais e no aproveitamento escolar dessa população^21^ ^.^ A identificação precoce de TMC e dos seus principais fatores de risco pode contribuir para intervenções específicas e melhor prognóstico.

No Brasil, alguns estudos avaliaram prevalência e fatores de risco para TMC na população adulta^13^ ^-^ ^15^ ^,^ ^25^ ^;^ entretanto, a literatura sobre TMC em adolescentes é escassa. Identificamos apenas um estudo transversal, de base populacional, conduzido entre adolescentes de 15 a 18 anos de Pelotas, RS, cuja prevalência de TMC foi de 28,8% e os principais fatores associados foram baixa escolaridade da mãe, fumo, comportamento sedentário e insatisfação com a imagem corporal^23^ ^.^

O presente estudo teve como objetivo descrever a prevalência de transtornos mentais comuns na população de adolescentes escolares brasileiros, segundo macrorregiões, tipo da escola e características sociodemográficas.

## MÉTODOS

Este estudo faz parte do Estudo de Riscos Cardiovasculares em Adolescentes (ERICA), um estudo transversal, nacional, de base escolar, conduzido em 2013-2014, com o objetivo de estimar a prevalência de síndrome metabólica, diabetes mellitus, obesidade, fatores de risco cardiovascular e de marcadores de resistência à insulina e inflamatórios em adolescentes de 12 a 17 anos que frequentam escolas em municípios brasileiros com mais de 100 mil habitantes.

Foram avaliados 74.589 adolescentes de 1.247 escolas em 124 municípios brasileiros. A população da pesquisa foi estratificada em 32 estratos constituídos por 27 capitais e cinco conjuntos de municípios com mais de 100 mil habitantes em cada macrorregião geográfica do País. Para cada estrato geográfico, as escolas foram selecionadas com probabilidade proporcional ao tamanho e inversamente proporcional à distância da capital. A amostra é representativa para o conjunto de municípios de mais de 100 mil habitantes em âmbito nacional, regional e para as capitais. Mais detalhes sobre o desenho de amostragem encontram-se em publicação anterior^28^ ^.^ Foram excluídos das análises, por não serem considerados elegíveis, adolescentes que não pertenciam à faixa etária de 12 a 17 anos, adolescentes grávidas e aqueles com deficiência física ou mental, temporária ou permanente. O protocolo do estudo foi previamente descrito.

Os dados foram coletados usando-se questionário autopreenchível e inseridos no coletor eletrônico de dados PDA (*personal digital assistant*). O questionário foi constituído por cerca de 100 questões divididas em 11 blocos: aspectos sociodemográficos, atividades ocupacionais, atividade física, comportamento alimentar, tabagismo, uso de bebidas alcoólicas, saúde reprodutiva, saúde bucal, duração do sono, morbidade física (autorreferida) e saúde mental.

No presente estudo, foram analisadas as seguintes características: sexo, idade, tipo de escola (pública ou privada), macrorregião geográfica (Norte, Nordeste, Centro-Oeste, Sudeste e Sul) e presença de TMC. A variável idade foi utilizada de forma categórica, considerando dois grupos: de 12-14 anos e de 15-17 anos.

Para avaliação de TMC, foi utilizado o *General Health Questionnaire*, versão de 12 itens (GHQ-12)^6^. Os escores dos itens individuais foram codificados como “ausente” ou “presente” (0 ou 1, respectivamente) e então somados; adolescentes com escore de três ou mais foram classificados como casos de TMC^7^. A versão brasileira, utilizada no presente estudo, foi submetida a estudo de validação, tendo uma entrevista psiquiátrica estruturada como padrão ouro e o mesmo critério de três ou mais para caso de TMC. Os resultados mostraram sensibilidade de 85,0% e especificidade de 79,0% e área abaixo da curva ROC (*Receiver Operating Characteristics*) de 0,87^17^.

Foram calculadas as prevalências e intervalos de 95% de confiança (IC95%) de TMC por sexo, idade, e tipo de escola, contemplando os âmbitos nacional, regional e por capitais.

As análises foram realizadas com o pacote estatístico Stata 14.0. As distribuições das características foram ajustadas segundo o desenho amostral, com o uso de rotinas estatísticas para amostragem complexa. A amostra do ERICA é uma amostra complexa^26^ ^,^ uma vez que emprega estratificação e conglomeração em seus estágios de seleção. Os pesos amostrais foram calculados pelo produto dos inversos das probabilidades de inclusão em cada estágio da amostra e foram calibrados considerando a projeção do número de adolescentes matriculados em escolas localizadas nos estratos geográficos considerados em 31/12/2013. Foi utilizado um estimador de pós-estratificação, que modifica o peso natural do desenho por um fator de calibração que corresponde à razão entre o total populacional e o total estimado pelo peso natural do desenho para o pós-estrato ou domínio de estimação considerado.

Todos os alunos participantes assinaram o termo de assentimento e trouxeram o termo de consentimento livre e esclarecido assinado pelos responsáveis (quando exigido pelo Comitê de Ética em Pesquisa local). O estudo foi aprovado pelos Comitês de Ética em Pesquisa da instituição da Coordenação Central do estudo (IESC/UFRJ – Processo 45/2008) e de cada estado brasileiro.

## RESULTADOS

Dos 102.327 adolescentes elegíveis cadastrados nas escolas selecionadas, participaram do estudo 74.589 (72,9%).

Quase um terço dos adolescentes de municípios de mais de 100 mil habitantes do Brasil apresentaram TMC. A prevalência foi mais elevada no sexo feminino e nos adolescentes mais velhos. As prevalências não apresentaram diferenças por macrorregião e tipo de escola (Tabela 1).

**Tabela 1:** Tamanho da amostra, população e prevalências de transtornos mentais comuns nos municípios com mais de 100 mil habitantes, segundo sexo, faixa etária, natureza da escola e macrorregião. ERICA, Brasil, 2013-2014.

Variável	Amostra	População estimada	Prevalência (%)	IC95%
Sexo				
Feminino	41.225	5.052.137	38,4	37,1-39,7
Masculino	33.364	5.095.563	21,6	20,5-22,8
Faixa etária				
12-14	34.141	5.348.201	26,7	25,8-27,6
15-17	40.448	4.799.499	33,6	32,2-35,0
Natureza da escola				
Pública	58.707	8.382.253	30,0	29,1-31,0
Privada	15.882	1.765.447	29,8	28,7-30,8
Macrorregião				
Norte	15.073	855.362	31,3	30,0-32,5
Nordeste	23.167	2.165.033	30,2	28,7-31,8
Centro-Oeste	9.727	778.010	31,7	30,4-33,0
Sudeste	17.080	5.153.506	29,3	28,0-30,7
Sul	9.542	1.195.789	30,4	28,6-32,2
Brasil	74.589	10.147.700	30,0	29,2-30,8

Com relação aos municípios, também não foram encontradas diferenças importantes, sendo que a maior prevalência de TMC foi observada em Manaus, AM, e a menor em Campo Grande, MS (Figura 1).

**Figura 1:** Prevalência e IC95% de transtorno mental comum nos municípios com mais de 100 mil habitantes por macrorregião e capital. ERICA, Brasil, 2013-2014.

As prevalências de TMC entre as meninas foi sempre maior do que entre os meninos, para todas as faixas etárias (Figura 2). Observa-se também uma tendência de aumento das prevalências de TMC com o aumento da idade.

**Figura 2:** Prevalência e IC95% de transtorno mental comum nos municípios de mais de 100 mil habitantes por sexo e idade. ERICA, Brasil, 2013-2014.

Nas análises estratificadas por sexo, faixa etária, tipo de escola e macrorregião, observa-se que a maior prevalência de TMC foi entre as meninas, na faixa etária de 15 a 17 anos e de escolas privadas da região Norte. A menor prevalência foi entre os meninos, na faixa etária de 12 a 14 anos, de escolas privadas da região Sudeste (Tabela 2).

**Tabela 2:** Prevalência e IC95% de transtornos mentais comuns em adolescentes por macrorregião, segundo natureza da escola, sexo e faixa etária. ERICA, Brasil, 2013-2014.

Variável	Brasil		Macrorregião									

			Norte		Nordeste		Centro-Oeste		Sul		Sudeste	
					
	%	IC95%	%	IC95%	%	IC95%	%	IC95%	%	IC95%	%	IC95%
Escola Pública												
Meninas												
12-14 anos	33,8	31,9-35,9	35,0	32,2-37,8	29,8	26,5-33,3	35,9	33,1-38,9	31,8	26,8-37,3	35,3	32,0-38,7
15-17 anos	42,3	40,0-44,6	44,9	43,1-46,8	42,5	40,0-45,1	44,7	42,1-47,4	40,7	36,4-45,1	41,7	37,6-46,0
Meninos												
12-14 anos	20,0	17,9-22,2	18,8	16,9-20,8	23,6	16,1-33,1	19,3	15,5-23,7	23,2	17,5-29,9	18,3	16,2-20,6
15-17 anos	24,0	22,4-25,6	25,4	23,3-27,6	24,5	21,8-27,4	25,8	23,5-28,3	26,7	22,7-31,2	22,6	20,0-25,3

Total	30,0	29,1-31,0	30,9	29,6-32,3	30,4	28,4-32,3	31,3	29,9-32,8	30,5	28,5-32,5	29,4	27,8-31,0

Escola Privada												
Meninas												
12-14 anos	36,4	33,3-39,5	39,1	33,0-45,5	35,3	32,4-38,3	38,2	34,2-42,4	32,8	26,5-39,8	37,2	31,7-43,1
15-17 anos	46,5	42,6-50,5	53,1	46,8-59,4	47,4	43,8-50,9	44,6	39,4-49,9	42,1	38,0-46,3	46,7	39,7-53,9
Meninos												
12-14 anos	16,3	14,6-18,2	20,4	15,6-26,2	18,1	15,4-21,2	20,0	14,3-27,1	21,5	15,5-29,0	13,0	11,0-15,4
15-17 anos	26,0	24,0-28,1	32,3	28,7-36,1	27,2	24,0-30,8	34,1	28,7-40,0	26,7	22,1-31,9	23,8	21,3-26,5

Total	29,8	28,7-30,8	35,0	31,6-38,6	29,7	27,9-31,6	33,3	30,8-36,0	29,6	26,5-32,8	28,8	27,2-30,4

## DISCUSSÃO

Este é o primeiro estudo epidemiológico conduzido no Brasil, com representatividade para os municípios de 100 mil habitantes ou mais em nível nacional, que avaliou dados de morbidade mental em adolescentes.

A prevalência geral destes transtornos foi elevada, sendo maior entre as meninas mais velhas (15-17 anos), de escolas privadas da região Norte, seguidas daquelas de escolas públicas da mesma região. As prevalências gerais por macrorregiões e por tipo de escola, entretanto, não mostraram diferenças marcantes.

Apesar de não dispormos de dados de saúde mental de adolescentes brasileiros, com representatividade nacional, a prevalência geral de TMC encontrada no ERICA (30,0%) foi semelhante àquela observada em estudo de base-populacional conduzido entre adolescentes de 15 a 18 anos residentes em Pelotas, município de médio porte no Sul do Brasil (28,8%)^23^ ^.^ A maior prevalência de TMC entre meninas (38,4%) quando comparada à dos meninos (21,6%), encontrada no presente estudo, também é consistente com os achados do estudo de Pelotas, em que a prevalência de TMC entre as meninas foi de 37,2% e a dos meninos de 19,9%. Estudo brasileiro conduzido em quatro municípios de quatro regiões do Brasil (Norte, Nordeste, Centro-Oeste e Sudeste) encontrou uma prevalência geral de 13,1% (nos últimos 12 meses) para pelo menos um transtorno psiquiátrico^22^. Essa prevalência é bem menor do que a encontrada em nosso estudo e reflete, provavelmente, algumas diferenças entre os dois estudos, a saber: 1) o fato de no ERICA ter sido utilizado um instrumento de rastreamento para transtornos mentais (GHQ-12), enquanto que no estudo conduzido por Paula et al^22^ ^,^ foi aplicado um instrumento para o diagnóstico de transtornos psiquiátricos (*Schedule for Affective Disorders and Schizophrenia for School-Age Children* – K-SADS-PL); 2) no ERICA, a faixa etária dos participantes foi de 12 a 17 anos, enquanto que, no estudo de Paula et al., os participantes tinham entre seis a 16 anos de idade; e 3) o ERICA é um estudo com representatividade nacional, conduzido entre 75.000 estudantes, enquanto, no estudo de Paula et al., a amostra foi apenas em quatro cidades. Além disso, no estudo de Paula et al., foi observada prevalência mais elevada (18,5%) de transtornos psiquiátricos (qualquer transtorno) na cidade localizada na região Centro-Oeste. No presente estudo, encontramos diferenças pouco marcantes nas prevalências de TMC por macrorregiões, sendo um pouco mais elevadas nas regiões Centro-Oeste e Norte.

Comparações com estudos internacionais são ainda mais difíceis, pelas diferenças de métodos, de tipos de transtornos e de faixas etárias entre os estudos. Entretanto, revisão de resultados de inquéritos de base populacional conduzidos em diferentes partes do mundo, mostra que, a despeito da variação substancial nos resultados, aproximadamente um quarto dos adolescentes experimentaram algum transtorno mental no ano anterior e um terço ao longo da vida^18^. Estudos também têm sido consistentes em apontar maiores prevalências de transtornos de ansiedade e humor entre as meninas, enquanto meninos apresentam maiores taxas de transtornos do comportamento e conduta^19^ ^,^ ^20^ ^,^ ^24^ ^,^ havendo variação com relação ao grau de renda e desenvolvimento dos países. Transtornos de conduta, de comunicação e transtornos globais do desenvolvimento são mais frequentes na fase inicial da infância, enquanto na adolescência são mais prevalentes os transtornos de conduta e depressão^20^ ^,^ ^24^.

Estudos mostram maior prevalência de transtornos entre adolescentes vivendo em países ou regiões menos favorecidos; porém, há inconsistência nos achados, refletindo prováveis diferenças de métodos. Giel et al.^5^ (1981) encontraram prevalências de transtornos mentais variando de 12,0% a 29,0% em crianças e adolescentes de cinco a 15 anos em quatro países de renda baixa ou média (Sudão, Filipinas, Colômbia e Índia). Estudo conduzido em Omã, na Arábia, com 5.409 adolescentes e adultos jovens de ambos os sexos, entre 14 e 23 anos, encontrou prevalência de 20,0% para pelo menos um diagnóstico psiquiátrico, de acordo com os critérios do *Diagnostic and Statistical Manual of Mental Disorders, Fourth Edition* (DSM-IV) e 32,7% para transtornos mentais leves (qualquer transtorno nos últimos 12 meses)^11^. Thabet e Vostanis^27^ (1998) reportaram prevalência de 21,0% de sintomas de ansiedade e transtornos relacionados entre crianças vivendo na Faixa de Gaza, comparáveis às encontradas por Kashani e Orvaschel^12^ (1990) na população de crianças e adolescentes dos EUA. Na Índia, meta-análise recente sobre a prevalência de transtornos mentais em crianças e adolescentes mostrou que, entre escolares de cinco a 15 anos, a taxa de prevalência de transtornos psiquiátricos foi de 23,3%^16^. Tais prevalências são menores do que a encontrada no presente estudo, em que foi usado instrumento para avaliação de TMC e não diagnósticos psiquiátricos, como os estudos acima descritos.

Este é o primeiro estudo que avaliou dados de saúde física e mental de adolescentes escolares, com representatividade para os municípios com mais de 100 mil habitantes, do Brasil, das macrorregiões e das capitais. Os TMC foram avaliados por instrumento padronizado e validado para a população de crianças e adolescentes^4^ ^,^ permitindo comparações com estudos nacionais e internacionais. Entretanto, deve-se considerar que o GHQ-12 é um instrumento de rastreamento sensível a mudanças psicológicas recentes, podendo levar a um número maior de falsos positivos, com sintomas transitórios de transtornos psicológicos, superestimando, portanto, a prevalência de TMC. Além disso, informações socioeconômicas mais detalhadas não foram incluídas nas análises e deverão ser consideradas em análises futuras.

Os transtornos mentais destacam-se como principais desafios a serem enfrentados pelos serviços de saúde. Muitas vezes, antes do diagnóstico formal de um transtorno psiquiátrico, já é possível encontrar indícios de sofrimento psíquico, na clínica, em adolescentes. Assim, a identificação precoce de TMC, bem como seus principais fatores de risco, pode ajudar na proposição de medidas de prevenção e controle mais específicos ao longo de todo o processo de desenvolvimento da adolescência.

O presente estudo, por estar inserido em um estudo maior, cujo principal objetivo é investigar fatores de risco cardiovasculares em adolescentes, incluiu, além da saúde mental, a mensuração de um número considerável de fatores, como características sociodemográficas, de estilo de vida, de morbidade referida e antropométricas e dados bioquímicos (em uma subamostra). Assim, espera-se que os resultados do presente estudo, que mostram elevada prevalência de TMC em crianças e adolescentes, forneçam evidência importante para estudos futuros que avaliem mecanismos comuns subjacentes aos transtornos mentais e às doenças cardiovasculares e, em uma perspectiva longitudinal, investiguem o papel das doenças cardiovasculares na ocorrência e persistência dos transtornos mentais. Tais estudos permitirão melhor compreensão dessa comorbidade, possibilitando o desenvolvimento de tratamentos e intervenções eficazes^10^.
